# Zeolite RHO Synthesis Accelerated by Ultrasonic Irradiation Treatment

**DOI:** 10.1038/s41598-019-51460-x

**Published:** 2019-10-21

**Authors:** Tiffany Yit Siew Ng, Thiam Leng Chew, Yin Fong Yeong, Zeinab Abbas Jawad, Chii-Dong Ho

**Affiliations:** 1 Department of Chemical Engineering, Faculty of Engineering, Universiti Teknologi PETRONAS, 32610 Seri Iskandar, Perak Malaysia; 2 CO2 Research Centre (CO2RES), Institute of Contaminant Management, Universiti Teknologi PETRONAS, 32610 Seri Iskandar, Perak Malaysia; 3 Curtin University Malaysia, Faculty of Engineering and Science, Chemical Engineering Department, 250CDT, 98009 Miri, Sarawak Malaysia; 4 Department of Chemical and Materials Engineering, Tamkang University, New Taipei City, 25137 Taiwan

**Keywords:** Synthesis and processing, Synthesis and processing

## Abstract

In recent years, there are increasing interest on applying ultrasonic irradiation for the synthesis of zeolite due to its advantages including remarkable shortened synthesis duration. In this project, the potential of ultrasonic irradiation treatment on the synthesis of zeolite RHO was investigated. Ultrasonic irradiation treatment time was varied from 30 to 120 minutes for the synthesis of zeolite RHO. The zeolite RHO solid samples were characterized with X-ray Diffraction (XRD), Field Emission Scanning Electron Microscopy (FESEM), Fourier Transform Infrared Spectroscopy (FTIR), Thermogravimetric Analysis (TGA) and nitrogen adsorption-desorption analysis. The application of ultrasonic irradiation treatment in this study has accelerated the synthesis of zeolite RHO where the synthesis duration has been significantly shortened to 2 days compared to 8 days required by conventional hydrothermal heating without ultrasonic irradiation treatment. Highly crystalline zeolite RHO crystals in truncated octahedron morphology were successfully formed.

## Introduction

Zeolites are crystalline microporous aluminosilicates with uniform molecular sized pore apertures, composed of oxygen, aluminum and silicon in its morphology. Their pores are able to selectively adsorb molecules and exclude the large sized molecules based on size exclusion and shape-selective basis^1,2^. According to International Zeolite Association (IZA) database, zeolites can be distinguished into large pore aperture zeolite with pore size of >7 Å (i.e. NaX, NaY, BEA, L-type, ZSM-12, etc), medium pore aperture zeolite with pore size of 5–6 Å (i.e. ZSM-5, ZSM-11, ZSM-57, silicalite, etc) and small pore aperture zeolite with pore size of 3–4 Å (i.e. RHO, DD3R, ZSM-58, SAPO-34, T-type, AlPO-18, etc)^3,4^. Zeolites are important in various fields due to the properties of well-defined pore structure as well as remarkable characteristics of high chemical and thermal stability^5–9^. Therefore, efforts to seek for efficient zeolite synthesis techniques that require short synthesis duration are one of the major interests of researchers nowadays.

Generally, synthesis of zeolite is performed via time consuming conventional hydrothermal heating at certain temperature and autogenous pressure. However, a survey of literature over the years has revealed a few major drawbacks. In conventional hydrothermal heating, it is difficult to control the nucleation in zeolites. Slow mixing rate and insufficient mixing between reactant particles cause supersaturation level of the zeolite precursor solution to increase to an unstable state and eventually lead to uncontrollable nucleation. This uncontrollable nucleation may become the main reason of larger crystal size and undesirable morphology of crystals being produced^10^.

The application of ultrasonic irradiation prior to hydrothermal heating appears to be a potential alternative in offering rapid synthesis for nano and microstructure particles^11^. Ultrasound waves are high frequency sound wave in the range of 20 kHz to 10 MHz where the frequency range falls above the human threshold of audibility^12^. Sonochemistry is the chemical effect of ultrasound arises from acoustic cavitation – the formation, growth and implosion of bubbles in liquid^11,13^. When ultrasound is transmitted through solid-liquid system, the alternating expansive (rarefaction region) and compressive (compression region) sound waves create variations in pressure and the position of molecules. The acoustic pressure at compression region is higher than ambient pressure when the molecules are being compressed while the acoustic pressure at rarefaction region is lower than ambient pressure when the molecules are being expanded. These variations produce bubbles or cavities in the liquid and accumulate the ultrasonic energy while growing to certain size until the maximum acoustic pressure at rarefaction has been reached^14^. The bubbles subsequently collapse and release large amount of shock wave energy that causes a stirring effect which results in more uniform and rapid mixing of reactants, thereby improving mass transfer in the reacting solution more efficiently compared to conventional agitation^10,15^. With enhanced mass transfer, the energy released is sufficient to initiate the nucleation and promote crystal growth in the solution, eventually changing the crystal morphology and size distribution^16^.

Ultrasonic irradiation is beneficial in reducing time of zeolite crystallization and producing well-defined zeolite crystals which are essential in industrial applications^17^. This is verified by Mubashir *et al*.^18^ where ultrasonic irradiation treatment was applied prior to conventional hydrothermal heating, thereby successfully reducing the synthesis duration of DD3R zeolite crystals from 25 days to 1 day while also decreasing the surface area of the crystals. Askari and Halladj^19^ successfully produced fully crystalline smaller-sized SAPO-34 crystals in 1.5 hours via sonochemical-assisted hydrothermal synthesis compared to 24 hours of conventional hydrothermal synthesis. High crystallinity of zeolite T particles were also successfully produced by Wahab and Yeong^20^ where the synthesis duration was shortened from 7 days to 2 days with 60 minutes of ultrasonic irradiation treatment.

Among various types of zeolites, zeolite RHO – a small pore aluminosilicate zeolite with pore opening of 3.6 Å × 3.6 Å and low Si/Al ratio of 2.5–3.0, can be one of the most promising candidates for various application^21^. The framework of zeolite RHO is constructed by body-centered cubic arrangement of α-cages linked via double 8-rings or truncated cubo-octahedra of silicon and aluminum tetrahedral^22^. Zeolite RHO is known to display flexible characteristic during the sorption process which enables itself to adapt to numerous sizes of cation and different shaped of adsorbate such as Na^+^, Cd^2+^, Sr^2+^, Rb^+^ or Ba^2+ ^^23,24^. Hence, zeolite RHO offers huge potential to be used in various fields. However, according to Liu *et al*.^25^, zeolite RHO requires a synthesis duration of 8 days via conventional hydrothermal heating without the addition of template agents. This long hydrothermal heating duration may have limited the application of zeolite RHO due to the high energy requirement and production cost in the synthesis process. Owing to that, it is of urgent need to explore an alternative technique to significantly shorten the synthesis duration of zeolite RHO.

In the current investigation, the synthesis of zeolite RHO via ultrasonic irradiation-assisted hydrothermal heating with different ultrasonic irradiation and hydrothermal heating durations were reported. The potential of ultrasonic irradiation treatment in the zeolite RHO synthesis was characterized by the aspects of crystallinity, morphology, functional groups, thermal stability and pore characteristic of the synthesized zeolite RHO. For comparison, the same synthesis process of zeolite RHO was conducted by conventional hydrothermal heating without ultrasonic irradiation treatment.

## Experimental

### Chemicals

The chemical reagents used to synthesize zeolite RHO were sodium aluminate (Sigma Aldrich, 53 wt% Al_2_O_3_, 47 wt% Na_2_O), colloidal silica LUDOX HS-40 (Sigma Aldrich, 40 wt%), cesium hydroxide (Sigma Aldrich, 50 wt% aqueous solution), sodium hydroxide (Fisher Scientific, >98%) and deionized water.

### Synthesis of the samples

The zeolite RHO samples were synthesized via ultrasonic irradiation-assisted hydrothermal heating. The precursor solution for zeolite RHO samples synthesis was prepared by following the composition of precursor solution reported by Mousavi *et al*.^4^. Sodium hydroxide was dissolved in deionized water under stirring by addition of cesium hydroxide and sodium aluminate consecutively. After complete dissolution, colloidal silica was added to the solution. The resultant precursor solution with molar composition of 3 Na_2_O : 0.4 Cs_2_O : Al_2_O_3_ : 10.8 SiO_2_ : 110 H_2_O was pretreated with ultrasonic irradiation for 30, 60 and 120 minutes respectively in an ultrasonic bath (Sonica 2400 EP S3) with a frequency of 40 kHz. The temperature of precursor solution pretreated with different ultrasonic irradiation time was measured by using thermocouple. Then, aging of the solution was conducted at room temperature for 24 hours. The aged solution was subjected to different hydrothermal heating durations of 0.5 to 2 days at 100 °C. Table 1 shows the matrix of different conditions for the preparation and synthesis of the samples in current study. The synthesized sample was centrifuged washed with deionized water, and dried overnight at 80 °C. For comparison, another zeolite RHO sample (S1) was synthesized as standard zeolite RHO sample via conventional hydrothermal heating for 8 days at 100 °C without ultrasonic irradiation treatment.

**Table 1:** Different samples synthesized under different conditions.

Sample	Ultrasonic Irradiation Time (mins)	Hydrothermal Heating Duration (days)
S1	—	8
S2	60	0.5
S3	60	1
S4	30	2
S5	60	2
S6	120	2

### Characterization of the samples

Morphology of the samples was observed with Field Emission Scanning Electron Microscopy (FESEM) (VP-FESEM, Zeiss Supra 55 VP). The XRD patterns of the samples was obtained via X-ray Diffraction (XRD) (Bruker D8 Advance) diffractometer with CuKα radiation in the 2θ range of 5–60°. Percentage of crystallinity of samples were calculated by using Eq. (1) at main XRD peaks with 2θ angles of 8.3°, 18.6° and 25.1°. The detailed equation for the calculation of percentage of crystallinity can be found as Supplementary Equation S1.

1 $$ \% \,{\rm{Crystallinity}}=\frac{\sum \,{\rm{Intensity}}\,{\rm{of}}\,{\rm{XRD}}\,{\rm{peaks}}\,{\rm{of}}\,{\rm{sample}}}{\sum \,{\rm{Intensity}}\,{\rm{of}}\,{\rm{XRD}}\,{\rm{peaks}}\,{\rm{of}}\,{\rm{standard}}\,{\rm{zeolite}}\,{\rm{RHO}}\,{\rm{sample}}}\times 100 \% $$

Framework vibration spectra of the samples was determined by Fourier Transform Infrared Spectroscopy (FTIR) (Perkin Elmer Frontier) within a wavelength from 4000 cm^−1^ to 400 cm^−1^. Thermogravimetric Analysis (TGA) of the samples was performed by heating from room temperature to 900 °C with a heating rate of 10 °C/min and flow rate of 20 mL/min under nitrogen atmosphere using Perkin Elmer Pyris. Nitrogen adsorption-desorption of the samples were conducted at 77 K by using Belsorp mini-II to measure the pore characteristics of the samples. The specific surface areas of the samples were determined by Brunauer-Emmett-Teller (BET) method.

## Results and Discussion

### Crystallinity of the samples

**Figure 1:**
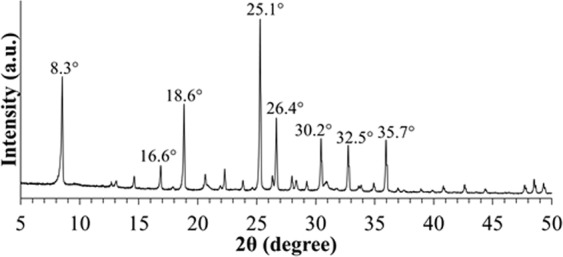
XRD patterns of sample S1.

Figure 1 shows the diffraction patterns of the synthesized sample S1 as standard zeolite RHO sample. After 8 days of conventional hydrothermal heating without ultrasonic irradiation treatment for sample S1, the observed characteristic diffraction peaks of zeolite RHO particles with the respective 2θ angles at 8.3°, 16.6°, 18.6°, 25.1°, 26.4°, 30.2°, 32.5° and 35.7° displayed similar XRD patterns with zeolite RHO reported in the literature, indicating highly crystalline zeolite RHO crystals have been successfully formed via conventional hydrothermal heating^25^.

**Figure 2:**
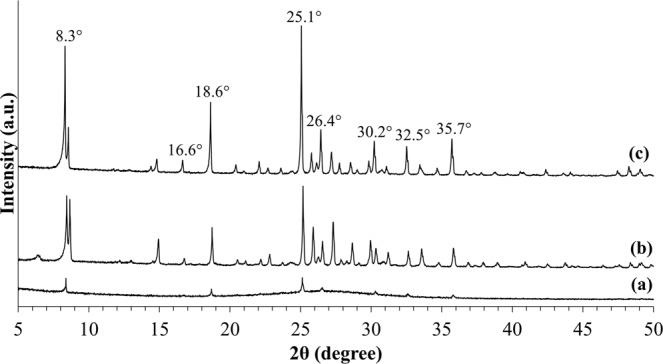
XRD patterns of the samples pretreated with 60 minutes of ultrasonic irradiation and followed by hydrothermal heating duration of (**a**) 0.5 days (S2), (**b**) 1 day (S3) and (**c**) 2 days (S5).

**Figure 3:**
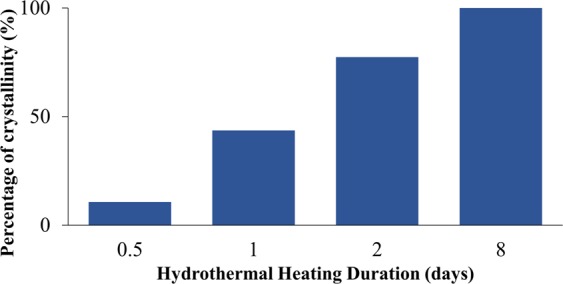
Changes in percentage of crystallinity for the samples pretreated with 60 minutes of ultrasonic irradiation followed by hydrothermal heating duration of (**a**) 0.5 days (S2), (**b**) 1 day (S3), (**c**) 2 days (S5) and (**d**) S1 (8 days of conventional hydrothermal heating without ultrasonic irradiation treatment).

The X-ray diffraction patterns of the synthesized samples pretreated with 60 minutes of ultrasonic irradiation followed by varied hydrothermal heating duration are demonstrated in Fig. 2. Figure 3 shows the effect of hydrothermal heating duration (at constant 60 minutes of ultrasonic irradiation treatment) on the percentage of crystallinity of the samples. By treating the precursor solution with ultrasonic irradiation for 60 minutes and followed by hydrothermal heating for only 0.5 days, zeolite RHO particles started to form with high amorphousity, as illustrated by the appeareance of very low intensity characteristic peaks for sample S2 in Fig. 2, which are close to the characteristic peaks of zeolite RHO sample S1. With the presence of ultrasonic irradiation treatment prior to hydrothermal heating, zeolite nuclei started to form at early synthesis time, implying an increase in zeolite nucleation rate which contributed to the shortened crystallization time for zeolite RHO synthesis^26^. According to Araki *et al*.^27^, no zeolite RHO particles was formed at all via hydrothermal heating up to 20 hours. However, by applying ultrasonic irradiation treatment for 60 minutes, followed by hydrothermal heating as short as 0.5 days only in the current study, it can be seen that zeolite RHO particles have already started to form. For sample S3 and S5, the obtained diffraction peaks are similar to the characteristic peaks of standard zeolite RHO sample S1, except the small diffraction peak difference at 2θ angle around 8.3°, which might be due to insufficient crystallization time for the respective samples. By prolonging the hydrothermal heating duration to 2 days, sharper diffraction peaks with higher intensity were observed as reflected by the main 2θ angles at 8.3°, 18.6° and 25.1° became more evident from sample S3 to sample S5. This indicates the increased crystallinity of zeolite RHO samples from 44% to 77% when the hydrothermal heating duration was increased from 1 to 2 days, as shown in Fig. 3.

**Figure 4:**
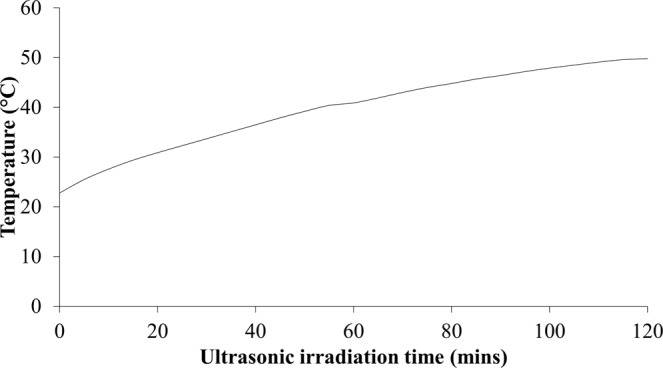
Temperature profile of the precursor solution pretreated with different ultrasonic irradiation time.

During ultrasonic irradiation treatment, the release of large amount of shock wave energy during collapsing of bubbles assists in the formation of free radicals and activates the reaction species in the reacting solution. Consequently, the mass transport in the reacting solution is enhanced as well as the reaction of hydrolysis and condensation which initiates nucleation process and zeolite crystal growth^28^. The release of shock wave energy during ultrasonic irradiation treatment was reflected by the increase in temperature of the precursor solution with increasing ultrasonic irradiation time as shown in Fig. 4.

An aluminosilicate zeolite mainly composed of oxygen, aluminum and silicon in its morphology where Si(OH)_4_ and Al(OH)_4_^−^ are the main reacting species in the formation of zeolite nuclei^28^. In an alkaline environment, the dominate silicate species will be anionic. The condensation reaction occurs through two reaction steps. The first step starts with the condensation of two silicic acid, Si(OH)_3_O^−^ to form Si-O-Si bond between two molecules while the second step is the removal of water molecule to form dimer species. At the same time, in a basic reacting medium, another condensation reaction also occurs through two reaction steps, which is the condensation of two stable Si(OH)_4_ and Al(OH)_4_^−^ species to form pentacoordinated intermediate species with Si-O-Al bond and followed by the removal of water molecule^29^. According to Augugliaro *et al*.^30^, application of ultrasonic irradiation in liquid produces free radicals H· and OH· from thermal splitting of water molecules during collapsing of bubbles. The thermal splitting mechanism of water molecules is demonstrated as below^28^:

$$\begin{array}{c}{{\rm{H}}}_{2}{\rm{O}}\to {\rm{H}}\cdot \,+\,{\rm{OH}}\cdot \\ {\rm{H}}\cdot \,+\,{\rm{H}}\cdot \to {{\rm{H}}}_{2}\\ {\rm{H}}\cdot \,+\,{\rm{OH}}\cdot \to {{\rm{H}}}_{2}{\rm{O}}\\ {\rm{OH}}\cdot \,+\,{\rm{OH}}\cdot \to {{\rm{H}}}_{2}{{\rm{O}}}_{2}\end{array}$$

**Figure 5:**
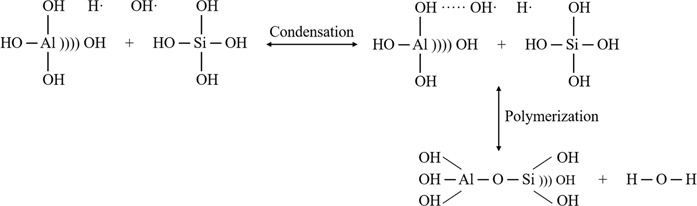
The proposed mechanism for the formation of aluminosilicate dimer^28^.

Figure 5 shows the proposed mechanism for the formation of aluminosilicate dimer reported by Pal *et al*.^28^. During sonochemical reaction, H_2_O functions as a proton donor and form hydrogen bond with the oxygen atom of SiOH. These strong oxidants and reductants are utilized and complete the formation of Si-O-Si and Si-O-Al bonds in the reacting medium^30^. By applying ultrasonic irradiation to the precursor solution, silica dissolution process will be enhanced in the reacting medium and thereby promotes the rate of polymerization-depolymerization for Si-O-Al bond in the system which contributes to the increased nucleation rate for crystalline zeolite RHO^31^. Without ultrasonic irradiation pretreatment, the processess of silica dissolution and Si-O-Al bond polymerization-depolymerization will be slowed down, resulting in low nucleation and crystallization rate which was observed in 8 days of conventional hydrothermal synthesis.

On the other hand, Cheng *et al*.^32^ reported a highly efficient and green radical route in accelerating the crystallization of zeolite. Sodium persulfate was used to produce highly active hydroxyl radicals which were able to catalyze the depolymerization of the aluminosilicate gel and polymerization of the aluminosilicate anions, subsequently accelerated the crystallization of the zeolite significantly^32^. Besides, Feng *et al*.^33^ investigated the crystallization of zeolite under ultraviolet irradiation condition. The hydroxyl free radicals generated under ultraviolet irradiation contributed in the acceleration of the nucleation stage during crystallization process of the zeolites^33^.

**Figure 6:**
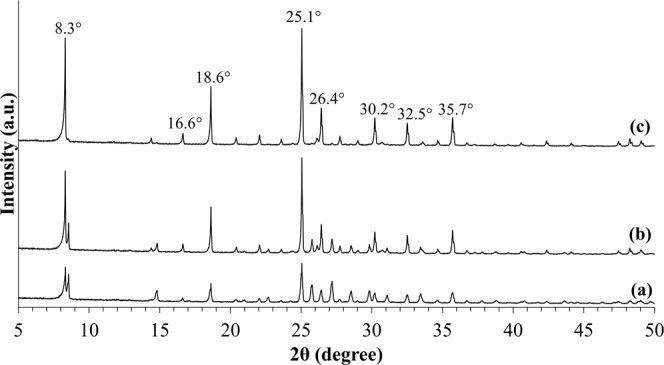
XRD patterns of the samples pretreated with varied ultrasonic irradiation time of (**a**) 30 minutes (S4), (**b**) 60 minutes (S5), (**c**) 120 minutes (S6) and followed by 2 days of hydrothermal heating.

**Figure 7:**
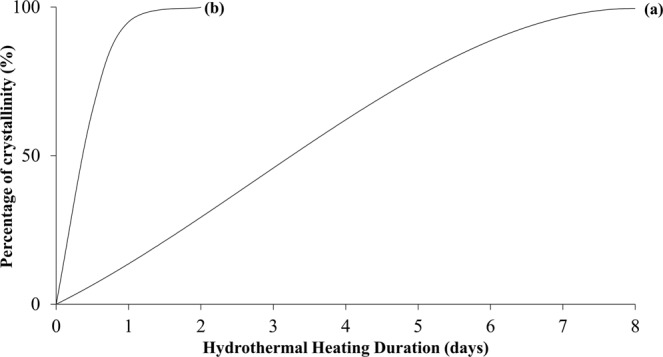
Crystallization curves of samples synthesized via (**a**) 8 days of conventional hydrothermal heating without ultrasonic irradiation treatment (S1) and (**b**) 120 minutes of ultrasonic irradiation pretreatment followed by 2 days of hydrothermal heating (S6).

Figure 6 shows diffraction patterns of samples S4, S5 and S6 which were pretreated with 30, 60 and 120 minutes of ultrasonic irradiation respectively, and followed by 2 days of hydrothermal heating. Difference in XRD peak at 2θ angle around 8.3° was observed for samples S4 and S5, compared to standard zeolite RHO sample S1. This can be corresponded to the insufficient crystallization time for the samples. By pretreating the samples with increasing ultrasonic irradiation time from 30 to 120 minutes prior to hydrothermal heating, the increase in intensity of diffraction peaks is clearly shown where the characteristic peak at 2θ angle of 8.3° became more evident from sample S4 to sample S6. By increasing the ultrasonic irradiation time, the percentage of crystallinity of the samples increases as well. It can be observed that complete crystallization was achieved by sample S6 which was pretreated by 120 minutes ultrasonic irradiation and followed by only 2 days of hydrothermal heating in current study. Figure 7 shows the crystallization curve for the samples synthesized without ultrasonic irradiation and with ultrasonic irradiation. The acceleration effect on the synthesis of zeolite RHO by applying ultrasonic irradiation treatment could be clearly observed from Fig. 7.

According to Araki *et al*.^27^, zeolite RHO particles can be formed in four main reaction steps. It was cited that amorphous aluminosilicates were formed upon dehydration and condensation between silica and alumina molecules via hydroxyl molecules in the solution at the first 3 hours. Then, crystals were started to grow and aggregation of amorphous aluminosilicates occurred after 3 hours. At 20 to 48 hours, crystallization has begun and amorphous aluminosilicates were slowly formed into crystalline zeolite RHO crystals with decreasing Na/Si ratio. Lastly, growth of crystals became gentle with increasing Na/Si ratio after 48 hours^27,34^. Conventionally, complete synthesis of zeolite RHO particles requires 8 days as reported by Liu *et al*.^25^. However, in the current study, the synthesis of zeolite RHO has been accelerated where the synthesis duration has been significantly shortened to 2 days by applying ultrasonic irradiation treatment for 120 minutes compared to the 8 days required by conventional hydrothermal heating without ultrasonic irradiation treatment.

### Microstructure and morphology of the samples

**Figure 8:**
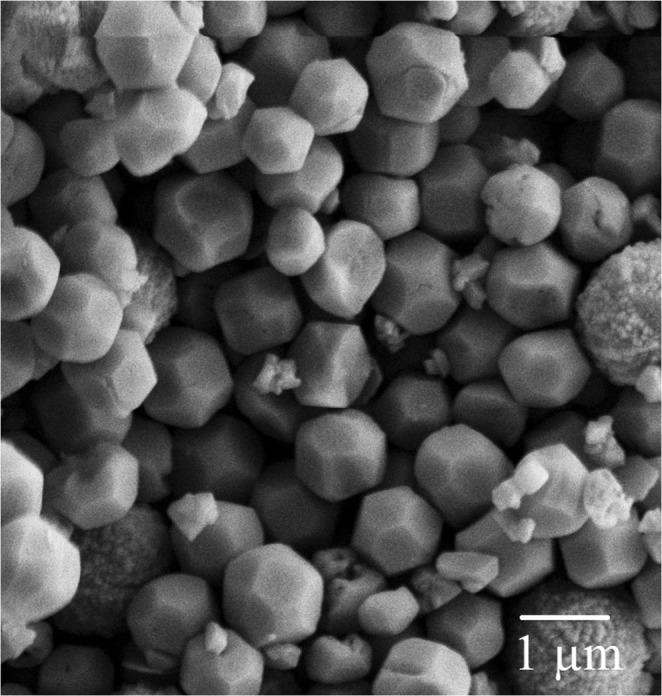
FESEM images of sample S1 synthesized in current study.

Figure 8 presents the microstructure and morphology of sample S1 synthesized in the current study. The zeolite RHO crystals formed in sample S1 exhibited a truncated octahedral shaped which agreed with the zeolite RHO crystals reported by Mousavi *et al*.^4^.

**Figure 9:**
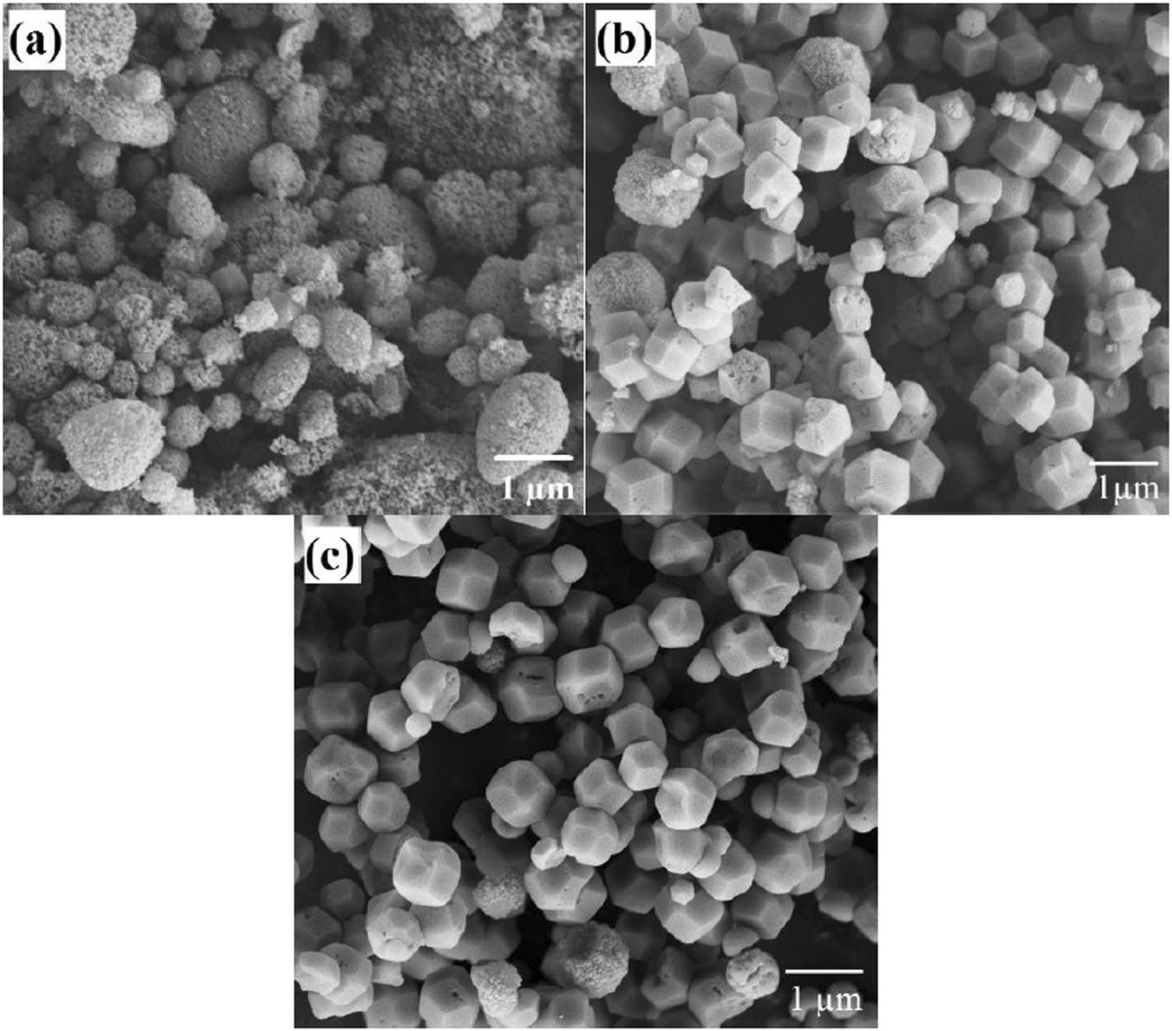
FESEM images of the samples pretreated with 60 minutes of ultrasonic irradiation and followed by hydrothermal heating duration (**a**) 0.5 days (S2), (**b**) 1 day (S3) and (**c**) 2 days (S5).

Figure 9 illustrates the morphology of samples synthesized via different hydrothermal heating duration but exposed to a constant 60 minutes of ultrasonic irradiation treatment. Based on Fig. 9, it can be clearly seen that highly amorphous aggregates were formed in sample S2. Small zeolite RHO crystals were also observed in sample S2 as supported by the low XRD peaks as shown in Fig. 2. As the hydrothermal heating duration was extended from 0.5 to 2 days, the amorphousity reduced and number of crystalline zeolite RHO crystals increased. The morphology and microstructure of samples S2, S3 and S5 suggest that the crystallinity of zeolite RHO particles increases with increasing hydrothermal heating duration from 0.5 to 2 days, which is in line with XRD pattern trend shown in Fig. 2.

**Figure 10:**
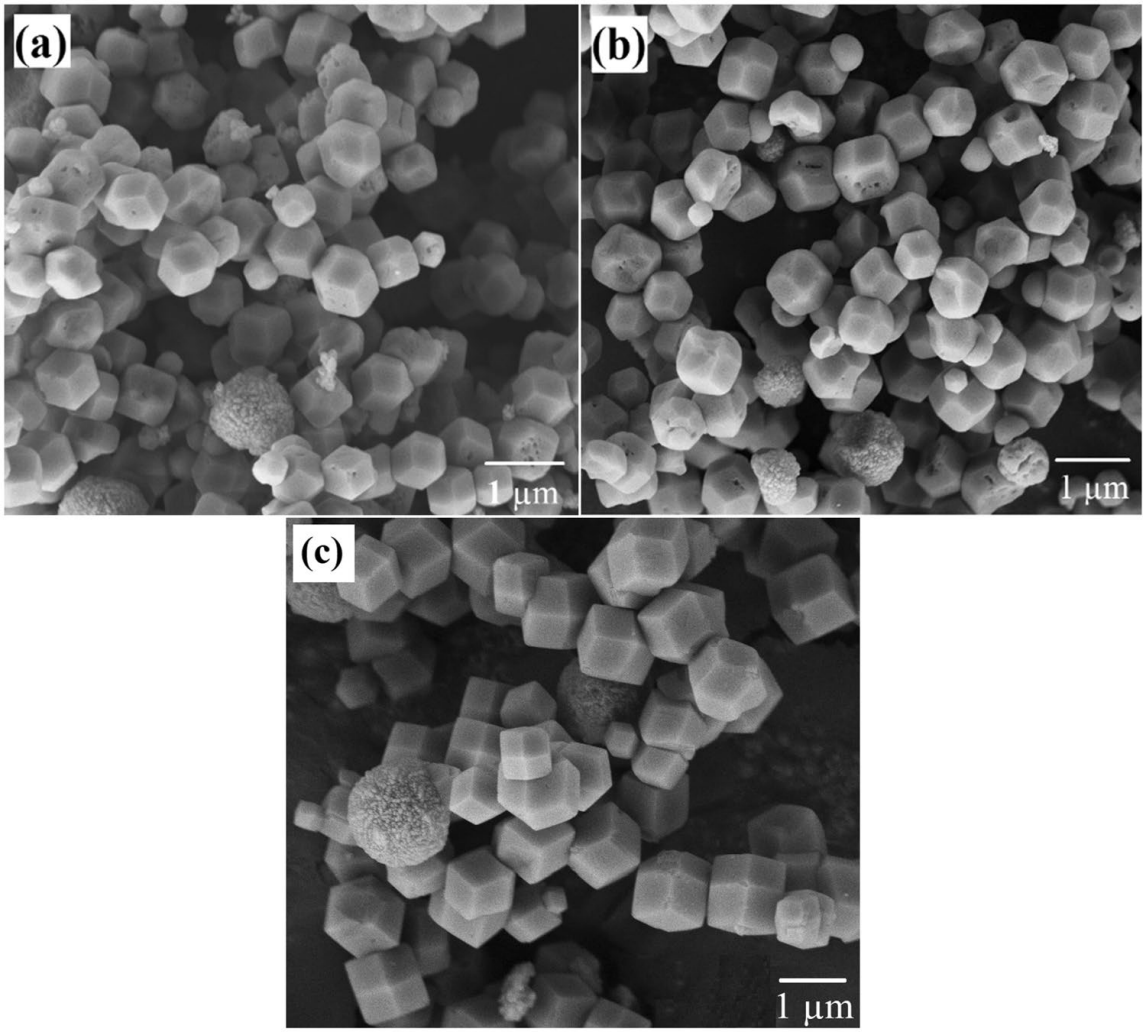
FESEM images of the samples pretreated with varied ultrasonic irradiation time of (**a**) 30 minutes (S4), (**b**) 60 minutes (S5), (**c**) 120 minutes (S6) and followed by 2 days of hydrothermal heating.

Figure 10 exhibits the morphology of samples S4, S5 and S6 which were pretreated with 30, 60 and 120 minutes of ultrasonic irradiation respectively, and followed by 2 days of hydrothermal heating. It is observed that truncated octahedral shaped zeolite RHO crystals are formed in all three samples. However, more spherical aggregates were found in sample S4 compared to those of samples S5 and S6 due to shorter ultrasonic irradiation time. These spherical aggregates with rough external surface require longer ultrasonic irradiation time to complete the nucleation and crystallization process and grow into crystalline solid crystals. The crystal size in samples S4, S5 and S6 were comparable to the crystal size of sample S1.

### FTIR spectrum of the samples

**Figure 11:**
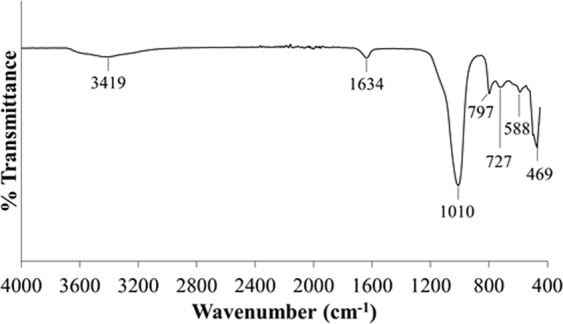
FTIR spectrum of sample S1.

The functional groups and molecular bondings of the samples were analyzed by FTIR and compared with the FTIR patterns of zeolite RHO particles reported by Flank^35^. Table 2 shows the FTIR characteristic bands displayed by Flank^35^, Byrappa and Kumar^36^ and Charkhi *et al*.^37^ whereas Fig. 11 displays the FTIR spectrum of sample S1. The FTIR spectrum of sample S1 agrees with the FTIR spectrum for zeolite RHO particles reported by Flank^35^. O-H stretching of adsorbed water molecules and O-H bending of lattice water were appeared as weak peaks at the frequency of 3419 cm^−1^ and 1634 cm^−1^ respectively. The peak at 1010 cm^−1^ corresponds to the vibrations due to the asymmetric of tetrahedral linkage in the zeolite RHO structure. Furthermore, the absorption band identified at 588 cm^−1^ indicates the presence of double 8-rings external linkage in zeolite RHO structure. The characterisitc band at 469 cm^−1^ is attributed to the TO_4_ (T = Si or Al) tetrahedral bending of zeolite RHO structure^35^.

**Table 2:** Type of bonding represented by FTIR spectrum for zeolite RHO particles^35–37^.

Characteristic band (cm^−1^)	Type of bonding
3650–3200	O-H stretching of adsorbed water
1700–1600	O-H bending of lattice water
1250–950	Si-O and Al-O tetrahedra asymmetrical stretching vibration
820–750	External linkage symmetrical stretching
720–600	Internal SiO_4_ group symmetrical stretching vibration
650–500	Double ring vibration
500–420	TO_4_ symmetrical stretching due to TO_4_ bending

**Figure 12:**
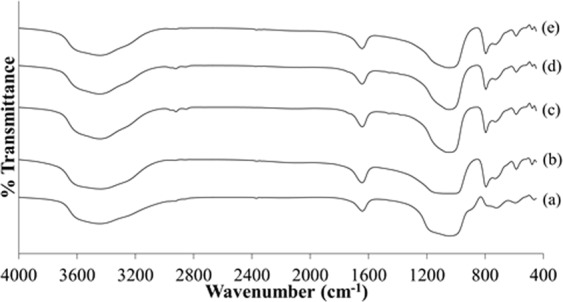
FTIR spectra of the samples (**a**) S2, (**b**) S3, (**c**) S4, (**d**) S5 and (**e**) S6.

FTIR spectrum of the samples (S2, S3, S4, S5 and S6) pretreated with 30, 60 and 120 minutes prior to 0.5, 1 and 2 days of hydrothermal heating were shown in Fig. 12. All the samples display FTIR spectrum with characteristic bands similar to sample S1 which confirms the presence of zeolite RHO particles in all samples. However, broad and less intense infrared spectrum that is attributed to T-O-T (T = Si or Al) tetrahedra asymmetrical stretching vibration was detected at 1010 cm^−1^ in sample S2 and S3. According to Rayalu *et al*.^38^, FTIR spectroscopy can also be used to predict the crystallinity of zeolites. For the samples pretreated with 60 minutes of ultrasonic irradiation and followed by increasing hydrothermal heating duration from 0.5 days (sample S2) to 2 days (sample S5), the crystallinity of the samples increased as can be seen when the characteristic band at 1010 cm^−1^ becomes sharper and narrower from sample S2 to S5.

### Thermogravimetric analysis of the samples

**Figure 13:**
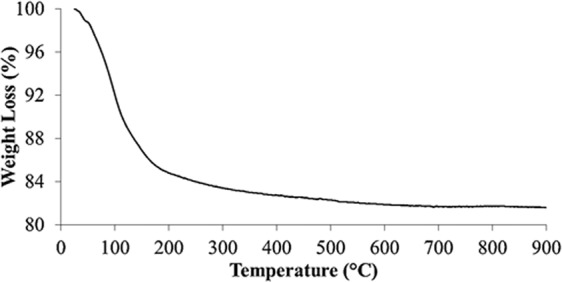
TGA curve of sample S1.

The TGA curve of the synthesized sample S1 performed under nitrogen is presented in Fig. 13 whereas Table 3 shows the weight loss of the synthesized samples under different conditions. From the data obtained, the synthesized samples in current study showed approximately 10.62–19.85% water loss up to 500 °C. 10.43–18.29% loss up to 200 °C may be attributed to the desorption of physically adsorbed water^34^. With further heating up from 200–500 °C, the weight loss of the synthesized samples of about 0.19–2.42% weight loss are due to the decomposition and removal of hydroxyl group in the as-synthesized zeolite samples^39^. The weight loss of the synthesized samples in current study are comparable with the work reported by Liu *et al*.^25^ and Chatelain *et al*.^34^.

**Table 3:** Weight loss of different samples synthesized under different conditions.

Sample	Weight Loss (%)	
	Desorption of Adsorbed Water at 30–200 °C	Decomposition of Hydroxyl Group at 200–500 °C
S1	15.28	2.42
S2	10.43	0.19
S3	13.68	1.16
S4	18.29	1.56
S5	14.63	1.45
S6	16.62	0.60

### Pore characteristics of the samples

Pore characteristics of the synthesized samples were summarized in Table 4. As can be observed in Table 4, sample S1 synthesized by 8 days of conventional hydrothermal heating exhibited BET specific surface area and pore volume of 33.05 m^2^ g^−1^ and 0.01977 cm^3^ g^−1^ respectively. On the other hand, sample S2 possessed the lowest BET specific surface area of 6.75 m^2^ g^−1^ and pore volume of 0.00103 cm^3^ g^−1^ among all samples. This is due to the formation of highly amorphous aggregates in sample S2 synthesized with insufficient hydrothermal heating duration of 0.5 days. By extending the ultrasonic irradiation pretreatment time from 30 to 120 minutes at constant hydrothermal heating duration of 2 days, crystallinity of samples increased and led to the rises in BET specific surface area and pore volume from sample S4 to sample S6 as shown in Table 4.

**Table 4:** Pore characteristics of the samples.

Sample	BET specific surface area, S_BET_ (m^2^ g^−1^)	Pore volume (cm^3^ g^-1^)
S1	33.05	0.01977
S2	6.75	0.00103
S3	20.60	0.00282
S4	22.26	0.00468
S5	24.22	0.00704
S6	44.36	0.00711

## Conclusion

Zeolite RHO has been successfully formed via ultrasonic irradiation-assisted hydrothermal heating as verified by XRD, FESEM, FTIR and TGA analysis. By applying ultrasonic irradiation treatment for 60 minutes, zeolite RHO started to form with hydrothermal heating as short as 0.5 days only. The application of ultrasonic irradiation treatment accelerated the synthesis of zeolite RHO and significantly shortened the conventional hydrothermal heating duration of 8 days to current hydrothermal heating duration of 2 days. Besides, ultrasonic irradiation assisted-hydrothermal heating with 120 minutes of ultrasonic irradiation treatment successfully produced truncated octahedron shaped zeolite RHO crystals with comparable crystal size with conventional hydrothermal heating. This current study has proved the potential of ultrasonic irradiation treatment in offering rapid synthesis of zeolite RHO.

## Supplementary information

Supplementary Information
