# Supplementary material for: Zeolite RHO Synthesis Accelerated by Ultrasonic Irradiation Treatment
Source: Sci Rep. 2019 Oct 21;9:15062. doi: 10.1038/s41598-019-51460-x (PMC6803675; doi:10.1038/s41598-019-51460-x)
Supplement: Supplementary file 1 — Supplementary Information [file 41598_2019_51460_MOESM1_ESM.pdf]

Supplementary Information

Zeolite RHO Synthesis Accelerated by Ultrasonic Irradiation Treatment

Tiffany Yit Siew Ng, Thiam Leng Chew, Yin Fong Yeong, Zeinab Abbas Jawad, Chii-Dong Ho

Supplementary Equation S1

$$\%\ \text{Crystallinity} = \frac{\sum \text{Intensity of main XRD peaks of sample}}{\sum \text{Intensity of main XRD peaks of standard zeolite RHO sample}} \times 100\%$$

$$= \frac{(\text{Intensity at } 2\theta = 8.3° + \text{Intensity at } 2\theta = 18.6° + \text{Intensity at } 2\theta = 25.1°) \text{ of sample}}{(\text{Intensity at } 2\theta = 8.3° + \text{Intensity at } 2\theta = 18.6° + \text{Intensity at } 2\theta = 25.1°) \text{ of standard zeolite RHO sample}} \times 100\% \quad (\text{S1})$$
